# Modeling Spatiotemporal Factors Associated With Sentiment on Twitter: Synthesis and Suggestions for Improving the Identification of Localized Deviations

**DOI:** 10.2196/12881

**Published:** 2019-05-08

**Authors:** Zubair Shah, Paige Martin, Enrico Coiera, Kenneth D Mandl, Adam G Dunn

**Affiliations:** 1 Centre for Health Informatics Australian Institute for Health Innovation Macquarie University Sydney Australia; 2 Computational Health Informatics Program Boston Children’s Hospital Boston, MA United States; 3 Department of Biomedical Informatics Harvard Medical School Boston, MA United States

**Keywords:** text mining, social media, public health

## Abstract

**Background:**

Studies examining how sentiment on social media varies depending on timing and location appear to produce inconsistent results, making it hard to design systems that use sentiment to detect localized events for public health applications.

**Objective:**

The aim of this study was to measure how common timing and location confounders explain variation in sentiment on Twitter.

**Methods:**

Using a dataset of 16.54 million English-language tweets from 100 cities posted between July 13 and November 30, 2017, we estimated the positive and negative sentiment for each of the cities using a dictionary-based sentiment analysis and constructed models to explain the differences in sentiment using time of day, day of week, weather, city, and interaction type (conversations or broadcasting) as factors and found that all factors were independently associated with sentiment.

**Results:**

In the full multivariable model of positive (Pearson r in test data 0.236; 95% CI 0.231-0.241) and negative (Pearson r in test data 0.306; 95% CI 0.301-0.310) sentiment, the city and time of day explained more of the variance than weather and day of week. Models that account for these confounders produce a different distribution and ranking of important events compared with models that do not account for these confounders.

**Conclusions:**

In public health applications that aim to detect localized events by aggregating sentiment across populations of Twitter users, it is worthwhile accounting for baseline differences before looking for unexpected changes.

## Introduction

### Background

Data from social media are increasingly being used in the digital phenotyping of individual users and the characterization of population-level behaviors to answer health-related questions [1-7]. Sentiment analysis is a broad class of methods used to detect opinions or mood from text. Although there are a range of approaches used in context-specific situations to detect positive and negative opinions about a topic [8-12], here we restrict the definition to include the general sentiment analysis methods used to detect mood. Sentiment analysis has also been used for applications in public health to evaluate reactions and attitudes to certain current events [13], health interventions such as vaccination [14], human mobility [15], and outcomes such as seasonal affective disorder and obesity [16-18].

When using sentiment analysis tools to observe or find signals of changes in the sentiment of a population, researchers must navigate the complicated interactions between the tools they use and the spatiotemporal and social factors that are known to modify mood and emotion. For example, the positive and negative affect measured by sentiment analysis has been shown to be associated with the time of day and day of week [19-21], weather [22-25], and the quality of social interactions [26].

Studies applying sentiment analysis to Twitter data have confirmed the periodicity of positive and negative affect by time of day and day of week [16,25,27-29]. However, the results and conclusions vary from study to study, and these differences may depend on the methods used to aggregate sentiment across sets of tweets or users, differences in the ways the investigators sampled the data, differences in the sentiment analysis algorithms or tools used, or because of challenges associated with validating results against external information. In comparison, studies examining variation in sentiment by geography or weather are relatively rare compared with those that measure temporal variation [30-34]. Studies that report analyses for social interactions on Twitter—tweets that mention, reply to, or quote other users—do not appear to have focused on measuring differences in the sentiment relative to tweets that broadcast a message [35].

### Objectives

The aim of this study was to construct simple models of positive and negative sentiment using time of day, day of week, interaction type, weather, or city as factors to understand how each of the different modifying factors might distort the results of public health studies that use sentiment analysis to study Twitter data. We then used the model and degenerate versions of the model to measure the magnitude of the differences between expected and observed sentiment over time and show how accounting for spatiotemporal differences affects the ranking of the importance of individual events.

## Methods

This study was an analysis of tweets posted by Twitter users in 100 cities. To address our aims, we aggregated sentiment scores for each hour in each of the 100 cities and constructed multivariable models to explain differences in the proportion of tweets, expressing positive or negative sentiment using city, interaction type, weather, time of day, and day of week as factors. We selected each of these factors because they have been shown to be associated with sentiment in past research and are relatively easily and accurately inferred from Twitter data.

### Twitter Data

We used the Twitter streaming application programming interface (API) to collect tweets between July 13 and November 30, 2017, without using any keywords. The retrieved tweets represent an approximate 1% sample of all tweets produced globally. Each tweet contains information about the user including name, location, tweet counts, follower counts, and following counts and the information about the tweet itself such as timestamp and the users it mentions.

Information in the tweet also provides information about whether it was a reply to a previous tweet, a retweet, or includes a link (quotes) to another tweet. We used this information to label each tweet as either broadcast (quotes, retweets, and tweets that do not mention other users) or social (replies and direct mentions of other users in the tweet).

### Location Data

Identifying the home locations of users on Twitter is a challenging task owing to the low number of posts with precise location information (geotags) and the need to parse user-defined location information using a gazetteer. Fewer than 0.5% tweets are geotagged, and fewer than 50% of Twitter users have provided useful home locations in their profiles [36]. To identify the location of the tweets from where it has been posted, we took the user-defined text from the location field in Twitter user profiles and used Nominatim, a gazetteer that returns a JavaScript Object Notation (JSON) object containing structured geographical information and a score associated with the confidence in the answer. Rather than filtering Nominatim results using a threshold on the confidence score, we found that Nominatim produces better results if we filter addresses based on type field of the return JSON object; therefore, we used type field in the returned JSON object to accept the top first address having type as city, county, village, suburb, hamlet, state, or country. This helped us to filter out other types of addresses without needing to use a specific threshold.

Not all Twitter accounts represent individuals; some are brands or organizations where tweets may be posted by humans or bots. Rahimi et al [37] used a simple but effective approach to removing *celebrities* in a study on location inference, in which they removed tweets from accounts that had more than 300,000 followers. After examining a set of Twitter users on either side of this threshold in our training data, we followed the same approach and removed all users with at least 300,000 followers.

### Timing Data

Past studies examining temporal patterns in sentiment on social media have found clear patterns [16,20,21,27]. However, those patterns vary substantially from study to study: some observed the most negative sentiment on Mondays and the most positive sentiment on Fridays or Saturdays. Some observed the strongest negative sentiment between 2 am and 5 am, whereas others observed the same between 8 pm and 11 pm.

As Twitter no longer includes a localized timestamp for users in the metadata of tweets, we used the identified location of the users posting the tweets to convert the timestamps of tweets from Universal Time Coordinated to local time. In what follows, all tweets are considered relative to the local time of the city in which the user is believed to be located.

### Weather Data

Past studies examining weather and sentiment on Twitter have produced variable results, but most observe one or more associations [31-33]. We collected hourly weather data for the top 100 cities using the API from the Open Weather website [38]. The information provided by the Open Weather website includes detailed weather information, such as temperature and humidity, and weather descriptions. We then mapped weather for each hour in each city to one of 7 values: clear, clouds, fog, haze, rain, snow, or storm.

### Sentiment Measures

Sentiment analysis of written text is a widely studied problem in natural language processing [39-41]. In this study, we have considered sentiment in a simple form—the presence of positive or negative affect—and applied SentiStrength [42], a widely used open-source Java library designed for sentiment analysis of tweets. It has been evaluated manually and compared with a range of advanced machine learning and statistical methods in several studies [42-44]. SentiStrength is a dictionary-based method, using a lexicon of words categorized as positive or negative with a score for its polarity and strength. For a given tweet, SentiStrength identifies the presence of sentiment terms from its lexicon and computes the sentiment of the text based upon the scores of the words found. SentiStrength produces 2 scores for each tweet, one indicating positive sentiment (from 1 to 5, least positive to most positive, respectively) and one indicating negative sentiment (from 1 to 5, least negative to most negative, respectively). As SentiStrength uses a score of +1 or −1 for neutral words, we considered scores from 2 to 5 for both positive and negative sentiments. In addition, as SentiStrength identifies positive and negative words independently, it is possible for a tweet to be labeled as having positive, negative, or both positive and negative sentiment.

We aggregated sentiment scores across a set of tweets using the proportion of tweets that have a positive sentiment score (a score from 2 to 5 in positive sentiment) or the proportion of tweets that have a negative sentiment score (a score from 2 to 5 in negative sentiment). Methods for aggregating scores across groups of tweets are important because they can influence the interpretation and lead to different conclusions. To aggregate sentiment scores, researchers have used counts, averages, proportions, ratios, and weighted averages [16,27,28,45-50]. Some have combined positive and negative scores to create a single measure [13,27,28,48,49], whereas others have kept positive and negative scores separate [46,47,51]. Following Scott et al [16], we used positive and negative sentiment scores separately because the positive and negative affect can coexist [52,53] and because when aggregated, a population can exhibit higher levels of both positive and negative sentiment at the same time. Thus, a low positive score indicates the absence of positive emotion across a set of tweets not the presence of negative emotion.

### Analysis and Modeling

In the first part of the analysis, we examined how each of the factors—interaction type, time of day, day of week, weather, and city—were associated with differences in the proportions of tweets that expressed positive or negative sentiment in a city in an hour. To do this, we constructed multivariable regression models using each of the factors individually and then in combination. We chose to use multivariable regression models because they are a simple way of capturing the baseline patterns of sentiment, and models built using individual factors and their combinations can be directly compared. For each model, we reported the r-squared value as a percentage, representing the percentage of the variance in sentiment that can be explained by each model.

In our evaluation of the models on unseen data, we then reported the correlation (Pearson r) between the values predicted by the model and the observed data in a set of testing data, distinct from the period of observation used to construct the models. These comparisons tell us how important each of the factors are as independent predictors of the sentiment for a city-hour pair and can provide guidance on which of the factors may be useful to control for when analyzing sentiment to detect changes or anomalies.

In the second part of the analysis, we have used the models constructed in the first part of the analysis as a baseline for detecting deviations from the expected proportions of positive and negative sentiment tweets per city per hour. The objective was to determine whether baseline differences in spatiotemporal and social factors would introduce biases in the detection of extreme deviations in sentiment that occur during major localized news events and if accounting for them in a baseline model could address these biases. To do this, we compared the expected and observed proportions of positive and negative sentiment tweets per city per hour using a chi-square test and then used the resulting *P* value as an indicator of the magnitude of the deviation.

Rather than defining an explicit threshold to label hour-city pairs as events or nonevents, we used the magnitude of the deviation in sentiment to rank all hour-city pairs in descending order based on the chi-square test. To make it easier to understand the expected frequency of the events, we defined a recurrence interval: the number of days of observation divided by the frequency of an event of that magnitude across the set of all cities in the analyses. For example, given 60 days of observation in the test period, a recurrence interval of 30 days is an event with a test statistic that was exceeded only twice during the 60 days. A recurrence interval of 1 day is an event with a magnitude that was exceeded 60 times in a 60-day period.

To characterize an event by its magnitude, we also needed to account for extreme sentiment that persisted for multiple hours or was expressed across multiple cities within a country. To do this, we merged events that produced significant differences between the observed and predicted number of positive or negative sentiment tweets and labeled them using the highest test statistic in the period. Similarly, we merged cities within a country if significant events occurred at the same time. As a result, hour-city pairs could be merged to produce day-city, day-country, or multi–day-country events depending on how many of the ranked deviations were traversed.

We then compared the events identified from the full model with the events produced by degenerate forms of the full model (eg, excluding city or interaction type as a factor). We used these differences to evaluate how the use of baseline spatiotemporal modeling affected the identification and ranking of extreme sentiment events. The expectation was that the degenerate forms of the models would introduce a bias in the distribution of events toward certain cities or times of day.

## Results

On average, we received 3.66 million tweets a day for 141 days, for a total of 507.60 million tweets from 27.61 million unique users. In the dataset, Twitter tagged 29.78% (151.21/507.60 million) as English language. Of these, 65.67% (99.30/151.21 million) had location information available in the users’ profiles.

**Figure 1:**
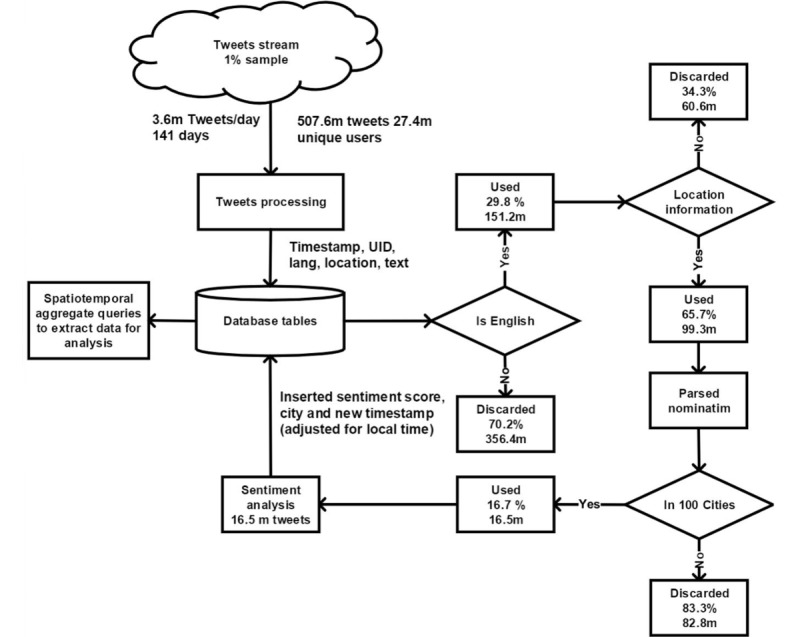
From 507.6 million tweets, 16.5 million were labelled as English language and attributed to users in 100 cities.

After removing celebrity/brand accounts, we ranked cities based on the total number of English language tweets posted by users with locations that the gazetteer was able to resolve. We identified the 100 cities with the highest numbers of English language tweets posted during the study period. These included 52 cities in North America (45 from the United States, 6 from Canada, and 1 from Mexico), 11 cities in the United Kingdom, 6 cities from Europe, 16 cities in Asia and Southeast Asia, 9 cities in Africa, 3 cities in Australasia, 2 cities from the Middle East, and 1 city in South America. We were able to resolve 16.61% (16.50/99.30 million) of the English language tweets to one of the 100 cities (Figure 1). We used these tweets as the basis for the study.

### Analysis of Spatiotemporal and Social Factors

**Figure 2:**
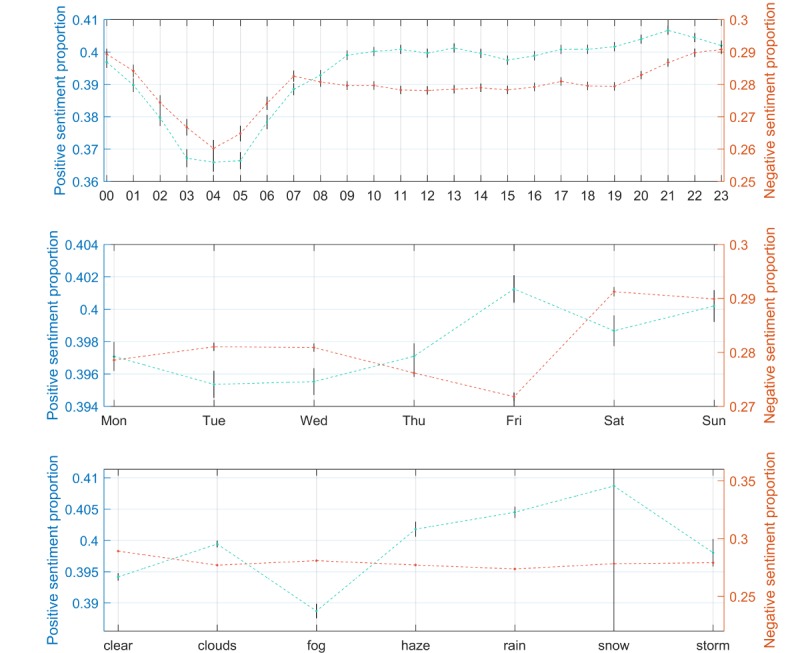
Observed proportions of positive and negative sentiment aggregated for all city-hour pairs by hour of the day (top), day of the week (center), and weather type (below). CIs are an indication of the number of city-hour pairs that contributed and the variability in proportion for that value. All values are categorical, so dotted lines are for visual interpretation only.

The training data used to construct the multivariable models comprised 8.39 million tweets from the first 81 days of data collection (July 13 to September 30, 2017). Of these, we found that 39.69% (3.33 million) expressed positive sentiment and 28.13% (2.36 million) expressed negative sentiment. Users across the 100 cities posted more tweets on Monday to Thursday and slightly fewer tweets from Friday to Sunday. The hour in which users were typically most active was between 12 noon and 1 pm (an average of 7652 tweets across the 100 cities), and users were least active between 4 am and 5 am (an average of 1745 tweets across the 100 cities). The number of tweets in each category of weather varied from snow (230 tweets) and storms (189,201 tweets) to cloudy weather (3,247,680 tweets). Relative to the average proportions of positive and negative sentiment, early morning hours exhibited lower proportions for both positive and negative sentiment, with the highest proportions of positive sentiment between 9 pm and 10 pm and highest rates of negative sentiment in the hours between 11 pm and 1 am, with an additional smaller peak between 7 am and 8 am (Figure 2). Fridays exhibited the highest proportion of positive sentiment and the lowest proportion of negative sentiment.

We constructed each model to estimate the proportion of tweets that expressed positive or negative sentiment in a city in an hour and have presented results based on the correlation between the estimated and observed proportions within the training data (Tables 1 and 2).

**Table 1:** Final model coefficient estimates for models of the proportion of tweets that exhibited negative sentiment in an hour.

Factor		Number of coefficients (number *P*<.05)	r-squared in the training period, %	Pearson r (95% CI) in the testing period
**Multiple factor models**				
	All factors	136 (108)	9.345	0.306 (0.301-0.310)
	Social, city, hour, day	130 (107)	9.338	0.306 (0.301-0.310)
	Social, city	101 (80)	8.831	0.297 (0.292-0.302)
	Hour, day	30 (26)	0.486	0.070 (0.065-0.075)
**Single factor models**				
	City	100 (81)	8.736	0.296 (0.291-0.300)
	Hour of day	24 (20)	0.298	0.055 (0.049-0.060)
	Day of week	7 (7)	0.191	0.044 (0.039-0.049)
	Weather	7 (5)	0.193	0.044 (0.039-0.049)
	Social proportion	2 (2)	0.010	0.010 (0.005-0.015)

**Table 2:** Final model coefficient estimates for models of the proportion of tweets that exhibited positive sentiment in an hour.

Factor		Number of coefficients (number *P*<.05)	r-squared in the training period, %	Pearson r (95% CI) in the testing period
**Multiple factor models**				
	All factors	136 (107)	5.584	0.236 (0.231-0.241)
	Social, city, hour, day	130 (107)	5.580	0.236 (0.231-0.241)
	Social, city	101 (85)	4.671	0.216 (0.211-0.221)
	Hour, day	30 (26)	1.330	0.115 (0.110-0.133)
**Single factor models**				
	City	100 (90)	3.732	0.193 (0.188-0.198)
	Hour of day	24 (21)	1.271	0.113 (0.108-0.118)
	Day of week	7 (6)	0.053	0.023 (0.018-0.028)
	Weather	7 (5)	0.170	0.041 (0.036-0.046)
	Social proportion	2 (2)	1.387	0.118 (0.113-0.123)

A model combining both temporal factors was significantly correlated with the proportion of tweets expressing negative sentiment (r=0.070; 95% CI 0.065-0.070). The association was stronger with the proportion of tweets expressing positive sentiment (r=0.115; 95% CI 0.110-0.133) and explained 5% of the variance. For both positive and negative sentiment outcomes, adding the day of the week to the hour of the day in the model produced a significant improvement in the model.

Positive and negative sentiment also varied by interaction type, where social tweets (tweets that mention or reply to another user) were much more likely to be expressions of positive sentiment relative to nonsocial tweets (tweets that do not mention or reply to another user). In hours where higher proportions of tweets were social interactions, the proportion of tweets that expressed positive sentiment were higher (r=0.118; 95% CI 0.113-0.123) and the proportion of tweets that expressed negative sentiment were lower (r=0.010; 95% CI 0.005-0.015) but this was a much weaker association. Adding the proportion of tweets that were social interactions as a factor in multivariable models made a significant improvement to the performance of the model in all cases.

**Figure 3:**
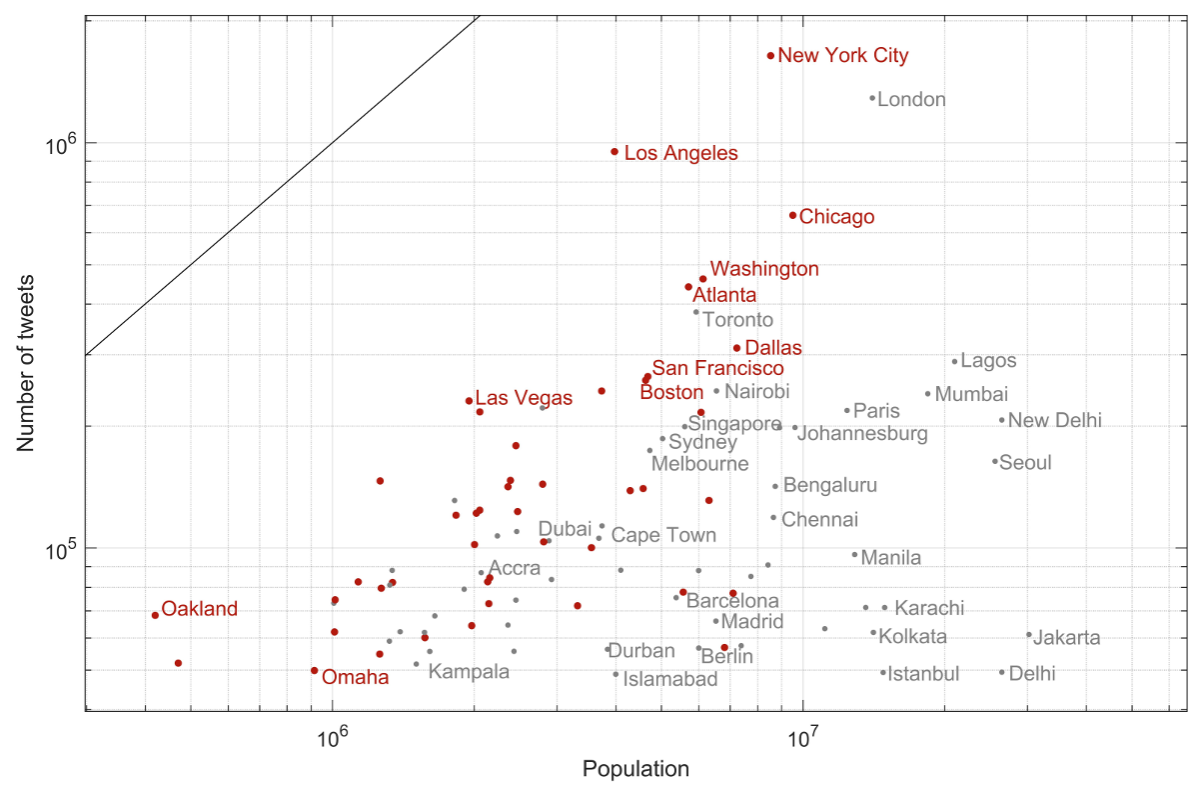
The number of tweets identified per city relative to the population of the city. Population data were manually collected from Wikipedia in December 2017, using the most recent metropolitan values available. Cities in the United States are highlighted in red and cities are partially labelled.

**Figure 4:**
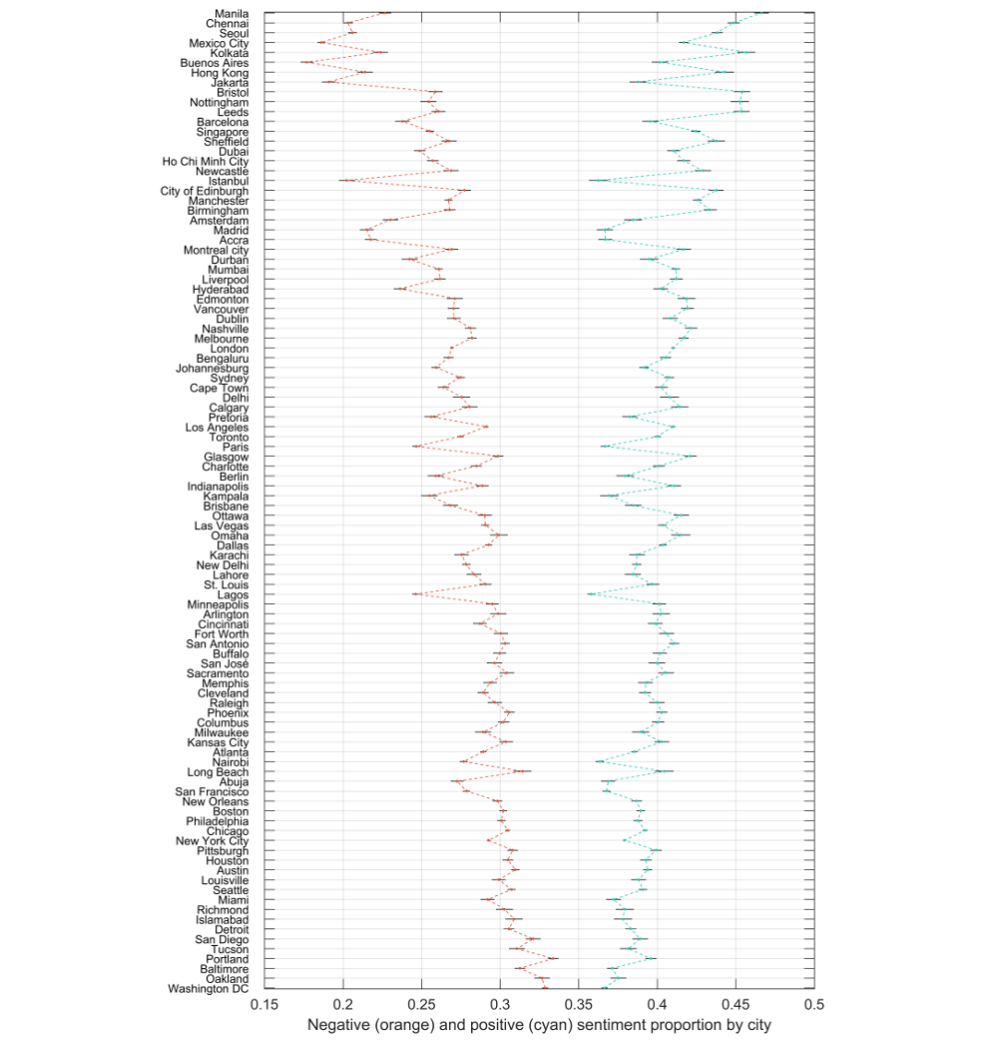
Sentiment by city in the training period, by proportion of positive (cyan) and negative (orange) sentiment tweets. Cities are ranked in decreasing order of the mean of the proportion of tweets with positive sentiment minus the proportion of tweets with negative sentiment.

The median number of tweets per city during the testing period was 48,974 and the number varied from 24,825 (Istanbul, Turkey) to 856,471 (New York City, United States). The numbers of tweets generally matched with the populations of the cities (Figure 3) and was lower for countries where languages other than English are used. Cities in the United States tended to have higher proportions of negative sentiment tweets and lower proportions of positive sentiment tweets (Figure 4). Models using only city information exhibited the strongest correlation with the proportion of positive and negative sentiment tweets in an hour compared with all other factors, explaining 8.73% of the variance in negative sentiment (r=0.296; 95% CI 0.291-0.300) and 3.70% of the variance in positive sentiment (r=0.193; 95% CI 0.188-0.198).

Weather exhibited weak associations with the proportions of tweets expressing positive (r=0.041; 95% CI 0.036-0.046) or negative sentiment (r=0.044; 95% CI 0.039-0.049). Its addition to the multivariable model including all other factors significantly improved the performance. However, as the coefficients for weather were orders of magnitude smaller than other factors such as city and social proportion, weather did not appear to be a useful addition to the baseline models used in the detection of variation in sentiment caused by exogenous factors.

### Detecting Deviations in City-Level Expression of Positive or Negative Sentiment

We then used the models constructed above to predict the expected sentiment in city-hour pairs constructed from a separate set of 8.02 million tweets from the following 60 days (October 1 to November 30, 2017). We found similar proportions of tweets expressing positive sentiment (3.20/8.02 million, 39.90%) or negative sentiment (2.28/8.02, 28.43%) as we found in the training data. For every hour-city pair, we determined the magnitude of localized deviations by measuring the difference between the expected and observed proportions of positive and negative sentiment tweets.

**Figure 5:**
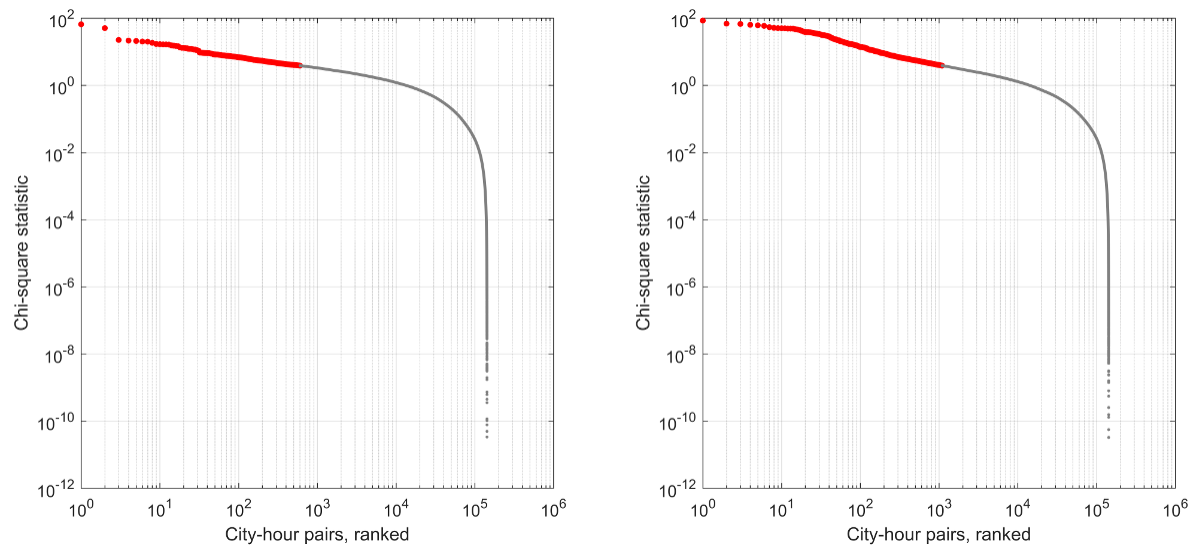
The set of all city-hour pairs for negative sentiment (left) and positive sentiment (right), ordered by decreasing the chi-square test statistic value. Note that there are thousands of city-hour pairs for which the test produces a P value under .05 (red). The recurrence interval for each city-hour pair is given by the value on the horizontal axis divided by the observation period in days (60 days).

Using the full model to identify unexpected deviations in the proportion of positive or negative sentiment tweets in the test period, we ranked events based on the magnitude of the deviation (Figure 5). As the number of events that might be considered important may vary depending on application, we have used the rank set of all city-hour pairs and traverse the list from the most extreme deviations to the least extreme deviations.

The top examples of localized deviations are listed in Table 3. We aggregated hour-city pairs across contiguous hours and cities wherever possible by reporting the most extreme deviation and merging any subsequent (less extreme) deviation that was on the same day (eg, extreme deviations in sentiment in the same direction on the same day in the same city are merged and reported as a day event) or cities in the same country (eg, 10 am in New York City and 10 am in Los Angeles is reported as 10 am in the United States). This was also extended to merge over both dimensions to report events by country and day. Where contiguous days reported events in the same direction, these events were merged as multi-day events.

**Figure 6:**
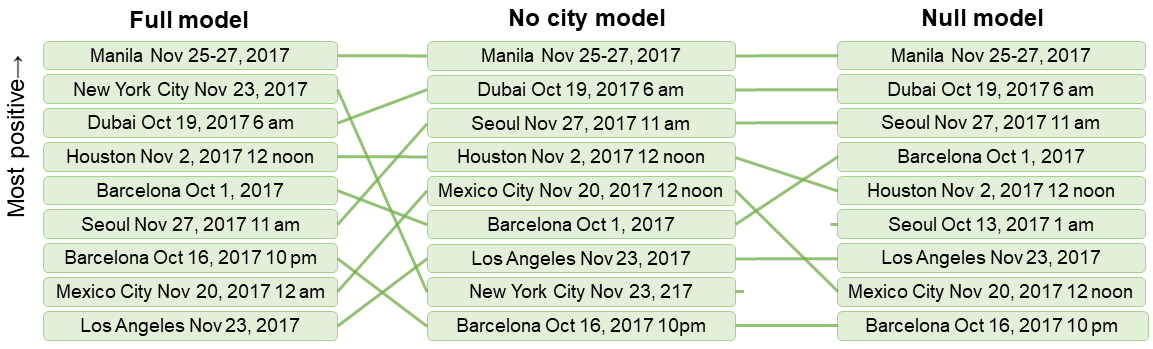
Most positive events for the 3 models aggregated where possible over hours, days, and cities. Note that compared with the full model (left), events from the United States tend to be moderated by the baseline tendency away from positive sentiment in the model without cities as factors (centre), and the null model (right).

After accounting for city-level differences in baseline proportions of positive and negative sentiment tweets, we found that the highest ranked events were distributed across 7 countries and could be retrospectively matched with major news stories that were specific to each of the cities. Using the degenerate models that do not account for city-level baseline differences, the United States accounted for a lower proportion of extreme positive events (Figure 6). This occurs because cities in the United States tend to exhibit higher rates of negative sentiment and lower rates of positive sentiment than cities in other countries. Models that do not take this baseline difference into account may overestimate the number of important negative events in the United States (which also has the effect of making violence in Barcelona or Nairobi seem less important) or underestimate the number of positive events in the United States (shifting down positive sentiment events such as Thanksgiving Day parade in New York City, New York or the World Series win in Houston, Texas).

**Table 3:** Examples of extreme city-level events with large deviations detected in sentiment.

Time and location	Percentage of negative sentiment tweets (% expected)	Percentage of positive sentiment tweets (% expected)	Recurrence interval (global; days)	Corresponding news event in the period
October 2, 2017 in the United States	49.56 (28.70)	31.30 (38.14)	>60	Coverage following Las Vegas shooting
November 25-27 in Manila	12.13 (22.91)	73.20 (45.67)	30	Miss Universe pageant
October 1-2, 2017 in Las Vegas	61.51 (30.72)	48.32 (40.52)	20	Shooting terror event at a music festival
October 1, 2017 in Barcelona	60.89 (23.78)	14.67 (39.56)	12	Voting for Catalonian independence
October 16, 2017 in Barcelona	67.41 (23.78)	17.8 (39.67)	10	Catalonian independence events
November 2, 2017 in Houston	14.41 (31.61)	56.60 (38.20)	8.6	Houston Astros win world series
November 23, 2017 in New York City	20.40 (29.01)	50.51 (37.40)	7.5	Thanksgiving Day parade
October 19, 2017 in Dubai	8.11 (25.01)	92.13 (39.02)	6	Diwali festival
October 27, 2017 in Nairobi	48.50 (26.52)	22.13 (37.32)	5.5	Riots following election
November 27, 2017 in Seoul	8.02 (21.01)	71.67 (43.30)	5	2 North Korean embarrassments
November 24, 2017 in London	35.50 (26.51)	47.12 (37.89)	4.6	False terror scare in Oxford Circus

**Figure 7:**
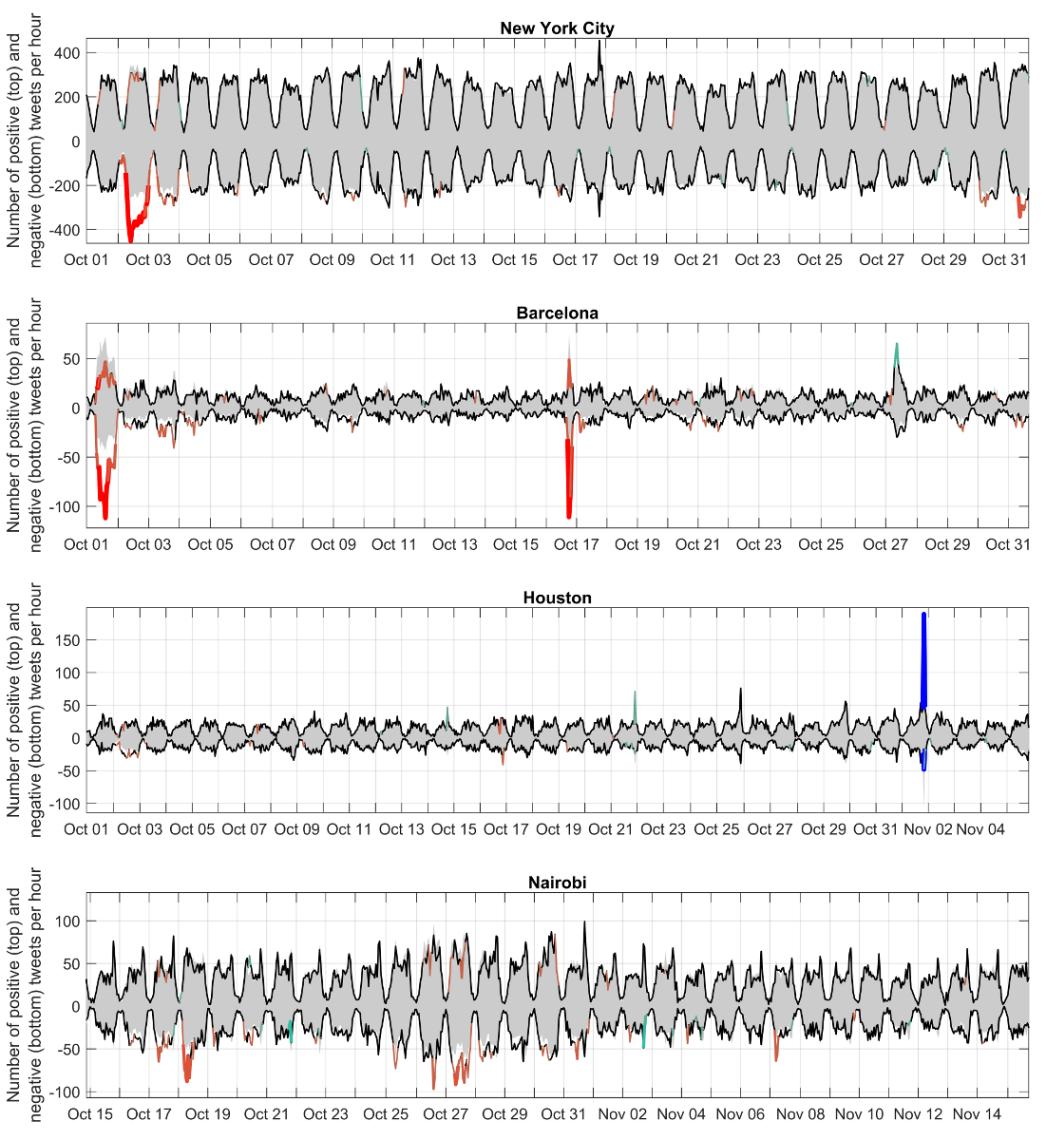
Examples of individual cities (New York City, Barcelona, Houston, and Nairobi) by the expected (gray areas) and observed (black and colored lines) sentiment. The color of the line indicates the magnitude of the deviation (darker red: more negative or fewer positive tweets; darker blue: more positive or fewer negative tweets).

From among the examples listed in Table 3, the visualization of the extreme events illustrates different types of deviations from the baseline (Figure 7). In each example, the expected baseline is the expected proportion of positive sentiment and negative sentiment tweets in an hour multiplied by the number of tweets from that city. Unexpected deviations occur when the observed number of positive or negative sentiment tweets is much higher or much lower than the baseline (in Figure 7, colored in red or blue). There were visible differences in the patterns indicating events that occur over a period of time (eg, riots after an election in Nairobi and a day of attempted voting in Barcelona) and events that occur within 1 or several hours (Houston Astros winning a baseball final). Other events not pictured include the outpouring of grief across multiple cities in the United States after a mass shooting, which decay more slowly over a period of days.

## Discussion

When applying sentiment analysis tools to Twitter data to characterize a population over time, it is useful to account for baseline spatiotemporal differences before attempting to detect deviations in mood. The first contribution of this study was to show that hour of day, day of week, the proportion of social tweets, the locations of the users posting the tweets, and the weather are each independently correlated with both positive and negative sentiment. Second, although these factors together account for less than 10% of the variance in positive and negative sentiment, ignoring them can affect the detection of unexpected deviations. Finally, we confirmed that in studies aggregating across populations (ecological designs), positive and negative sentiment can rise and fall independently and aggregating them into a single measure may mean losing important information that helps characterize the mood of a population.

### Comparisons With Past Literature and Implications

A range of studies have applied sentiment analysis tools to social media data to examine changes in mood or emotion in relation to current events, weather and season, or circadian and daily rhythms. Our results extend these analyses to demonstrate the relative importance of each of these factors.

We found that the time of day and day of week were more closely correlated with positive sentiment than with negative sentiment. For positive sentiment, models built using these temporal factors typically explain less of the variance than models that used social interactions and cities as factors. Previous studies investigating hourly and daily patterns of sentiment on Twitter vary in structure from cohort designs, where individual users are followed [16,54], to ecological designs where signals from a population are aggregated [27,29,55]. The results of these studies and the conclusions they draw appear to be related to design choices including the tools used to measure sentiment and the methods used to aggregate measures of sentiment across populations.

The results of the study are consistent with previous studies that have found associations between weather and sentiment on Twitter [31-33]. Despite the observed independent correlations between weather and sentiment, weather explained little of the variance in positive or negative sentiment. These results should not be confused with seasonal variation in weather or sunlight; our results did not extend across a full range of seasons, and other studies have examined the use of Twitter data for its potential to observe seasonal affective disorder [16,17]. Mitchell et al [30] examined the geography of happiness in 373 cities in the United States using Twitter data and found that happiness was correlated with socioeconomic status and health-related census data, among other factors. We found that negative sentiment was more common and positive sentiment less common in tweets from many cities in the United States and suggest that future research in the area would benefit from studying international differences in sentiment associated with culture and patterns of living and working that might influence the expression of sentiment on social media.

Tweets that involve social interactions on Twitter (typically replies and mentions) are common in applications of network science. Our results show a strong positive correlation between the proportion of social interactions in a city in an hour and positive sentiment and a weak correlation with negative sentiment. Future applications that couple network analysis with sentiment tools may benefit from recognizing and potentially accounting for the differences between tweets that are social in nature, relative to those that are broadcasting information.

Twitter and other social media platforms offer the opportunity to undertake naturalistic studies of human behaviors at unprecedented scales [56-59]. However, studies in the area are at risk of producing incomparable results and inconsistent conclusions if sampling methods vary in ways that skew toward certain locations or certain times of the day or week. Practitioners in the area are already aware of the risks of selecting only geotagged tweets [60], but the spatiotemporal differences we highlight here are typically not discussed or accounted for in applications that use Twitter data to answer public health questions.

### Limitations and Future Work

The study has several limitations. First, Twitter users represent a biased sample of countries and a biased sample of the population within countries [60-64], and we did not infer the demographics nor apply any reweighting methods to adjust for differences between the users posting English language tweets and the demographics of the cities we examined. Furthermore, users who include enough biographical information to be located within a city may represent a biased subset of the overall Twitter population, and we did not use location inference methods that take advantage of location-indicative words or social network structure [65-68] because these could introduce further sampling biases (eg, the overlapping of words in the dictionary and those that are useful in predicting a location). For these reasons, the study only captures deviations that might be expected to be important to population-level (epidemiological) studies.

Second, we used SentiStrength as a measure of sentiment and did not consider alternatives, sentiment in languages other than English, or ensembles combining multiple tools [69-72]. We think our use of SentiStrength is justified because it is a commonly used tool in studies in public health and has been examined for sentence-level sentiment and on individual tweets previously [42,44,45]. Although we did not test multiple sentiment detection methods to confirm, we expect that the need to account for baseline spatiotemporal differences is likely to be useful across all other sentiment detection approaches.

Third, certain events are less localized and affect multiple cities or even multiple countries and others may extend across many hours, days, or weeks. Methods for dealing with the spatiotemporal granularity of these events would be a useful addition to the sets of methods used in analyses of sentiment (or other measures that can be observed in social media datasets). Real-time event detection on Twitter is an active area of research [73,74], and our aim was not to add to this literature. Rather, we sought to develop a way to improve the robustness of observational studies that use sentiment analysis of Twitter to make sense of how populations react to real-world events.

Finally, we selected a set of factors that were known to be associated with sentiment on Twitter and used a relatively simple approach to modeling their associations. Other user-level factors and more sophisticated models may improve our ability to account for baseline differences in sentiment, including heterogeneity of individual-level differences that are apparent at population-level scales. For example, other factors that could have been included are gender, age, and number of followers; and other modeling pipelines might consider feature selection or dimensionality reduction and cross-validation techniques to avoid overfitting and improve generalization.

### Conclusions

In this study we showed that in applications that use population-level measures of sentiment on Twitter, it is useful to account for baseline differences in sentiment by time of day, day of week, location, weather, and interaction type. Doing so could improve the accuracy of methods that use sentiment to detect localized events or changes in mood. The first contribution of this research is the consistent evaluation of a broad set of factors—making it easier to compare the importance of location, time, and social interactions on positive and negative sentiment. The second contribution is the use of these factors to construct a simple and interpretable model of the expected variation in positive and negative sentiment on Twitter.

## Abbreviations

API: application programming interface
JSON: JavaScript Object Notation
